# A Security Analysis of the 802.11s Wireless Mesh Network Routing Protocol and Its Secure Routing Protocols

**DOI:** 10.3390/s130911553

**Published:** 2013-09-02

**Authors:** Whye Kit Tan, Sang-Gon Lee, Jun Huy Lam, Seong-Moo Yoo

**Affiliations:** 1 Department of Ubiquitous IT, Division of Computer & Information Engineering, Dongseo University, Busan 617-716, Korea; E-Mails: blueppp@gmail.com (W.K.T.); timljh@msn.com (J.H.L.); 2 Department of Electrical and Computer Engineering, The University of Alabama in Huntsville, Huntsville, AL 35899, USA; E-Mail: yoos@uah.edu

**Keywords:** hybrid wireless mesh protocol, secure routing, wireless mesh network, 802.11s, sensor network

## Abstract

Wireless mesh networks (WMNs) can act as a scalable backbone by connecting separate sensor networks and even by connecting WMNs to a wired network. The Hybrid Wireless Mesh Protocol (HWMP) is the default routing protocol for the 802.11s WMN. The routing protocol is one of the most important parts of the network, and it requires protection, especially in the wireless environment. The existing security protocols, such as the Broadcast Integrity Protocol (BIP), Counter with cipher block chaining message authentication code protocol (CCMP), Secure Hybrid Wireless Mesh Protocol (SHWMP), Identity Based Cryptography HWMP (IBC-HWMP), Elliptic Curve Digital Signature Algorithm HWMP (ECDSA-HWMP), and Watchdog-HWMP aim to protect the HWMP frames. In this paper, we have analyzed the vulnerabilities of the HWMP and developed security requirements to protect these identified vulnerabilities. We applied the security requirements to analyze the existing secure schemes for HWMP. The results of our analysis indicate that none of these protocols is able to satisfy all of the security requirements. We also present a quantitative complexity comparison among the protocols and an example of a security scheme for HWMP to demonstrate how the result of our research can be utilized. Our research results thus provide a tool for designing secure schemes for the HWMP.

## 1. Introduction

A WMN combines the advantages of wireless connections with the advantages of a mesh topology. A WMN provides better mobility, a lower cost of deployment, easier network expansion, and robust connections. A WMN is suitable for many applications, including broadband home networking, enterprise networking, building automation systems, and health and medical systems [1,2]. Because the WMN was developed first for industrial purposes, each company had its own proprietary technology, and no industrial standard existed for WMNs. As a result, different WMN devices were incompatible with each other. In 2004, the Institute of Electrical and Electronics Engineering (IEEE) started the 802.11s Task Group (TG) to develop a standard for WMNs [3]. There are many standards related to WMN implementation, including 802.11s (Wi-Fi), 802.15.1 (wireless personal area network (WPAN) Bluetooth), 802.15.4 (WPAN ZigBee), and 802.16a (Worldwide Interoperability for Microwave Access (WiMAX)). The standard for 802.11s was finalized in July 2011 [3].

The widely deployed 802.11 wireless local area network (WLAN) infrastructure can be connected to a wireless sensor network (WSN) both to save on costs for deploying a new network and to utilize the advantages of 802.11 technologies [4]. A WMN can act as a scalable backbone by connecting separate sensor networks and even by connecting WMNs to a wired network. Initial research reported on the interconnection between WMN and WSN [5]. Subsequently, concerns over the power consumption related to 802.11 were also eliminated with power-efficient standards for the 802.11. Now, a sensor device is able to run for 5 to 10 years on a single AA battery [4]. Such a sensor device can then be used as a gateway for the WSN. The data rate for 802.11 is also very high compared to the common technologies used in a WSN, such as ZigBee, Bluetooth, or 802.15.4 [6]. IEEE 802.11s can be used to form the mesh topology or bridge networks for the WSN.

802.11s allows Wi-Fi devices to organize themselves and configure the network topology automatically. Wi-Fi devices with mesh functionality are referred to as mesh stations (or mesh STA). Mesh STAs that are far apart can still communicate with each other by utilizing wireless routing, where data packets are transmitted through intermediate mesh STAs. Hybrid Wireless Mesh Protocol (HWMP) is the default routing protocol for 802.11s, which must be implemented in layer 2. Thus, each 802.11s node is considered to be a layer 2 device. While the working principles and operations of HWMP have been defined in the drafted standard, the security features for the HWMP are not discussed in the drafted standard [7]. It is possible to treat the HWMP frames as normal management frames and to protect them with the same protocols as defined in 802.11w [8], but those protocols do not consider a multi-hopping environment.

In this paper, we investigate how to protect routing messages, rather than data messages. In general, routing messages are sent to the immediate neighbors, at which point the neighbors process, possibly modify, and resend the messages. Therefore, some parts of these messages may be changed by the intermediate nodes during their propagation over the network. The most commonly changed parts of the routing message include the hop count and the metric of the path requested or provided. In terms of network security, we do not trust the intermediate node [9]. Thus, for the security of routing messages, the two types of message parts (*i.e.*, the mutable and non-mutable fields) have different security requirements. The mutable fields (MFs) should be updated according to the routing rule as the message is propagated along the path. Each node requires a cryptographic scheme to detect illegal mutable information in the message. For the security of non-mutable fields (NMFs), the data integrity and data-origin authentication service for the field must be provided between the source and destination nodes.

Some prior research exists on securing the layer-3 routing protocols for a WMN [10]. Secure ad hoc on-demand distance vector (SAODV) routing [9] is a secure variant of the ad hoc on-demand distance vector (AODV) routing. Authenticated routing for *ad hoc* networks (ARAN) [11] uses public key cryptography to ensure the integrity of the routing messages. For example, Ariadne [12] ensures a secure on-demand source routing. The Secure Route Discovery Protocol (SRDP) [13] uses a one-way hash function for route requests and public key signatures for route reply messages. All of these protocols provide end-to-end security between the source and destination nodes, and each has it own pros and cons [10,14]. Ning *et al.* [15] studied some insider attacks against the AODV routing protocol, which is very similar to the reactive mode of HWMP. The authors successfully achieved the attacking goals for routing protocol, including route disruption, route invasion, node isolation, and resource consumption, by misusing the protocol messages, route request (RREQ), route reply (RREP), and route error (RERR) messages. Their misuse actions are message drop, modifying and forwarding, forge replying, and active forging. Both the MFs and NMFs are possible message modification targets. Their attacks are effective to the reactive mode of the HWMP.

Prior research also exists on securing the layer-2 routing protocol HWMP. The secure HWMP (SHWMP) [10] secures HWMP frames from external attacks. However, SHWMP does not address internal attacks. SHWMP provides integrity service for the MFs and confidentiality service for the NMFs in a point-to-point link. This protocol does not provide integrity assurance from the source node to protect against non-mutable field modification attacks. This protocol also does not have any security schemes for the path error (PERR) HWMP routing protocol frames.

Ben-Othman *et al.* [16] proposed a security mechanism based on the identity-based cryptography (IBC) to secure HWMP. Hereafter, we denote this security mechanism as IBC-HWMP. A media access control (MAC) address is used as the identity for all mesh STAs. A private key is used to sign the MF such that the integrity of the MF is protected. Like SHWMP, the IBC-HWMP protocol also does not provide integrity assurance from the source node to protect against non-mutable field modification attacks. This protocol does not have any security schemes for PERR or root announcement (RANN) HWMP routing protocol frames.

In terms of the design process of a secure scheme for a routing protocol, the vulnerabilities analysis of the protocol is the first step. The second step is setting up suitable security requirements to protect against the identified vulnerabilities. The last step is designing a secure scheme that satisfies the requirements. The existing secure schemes have not been designed using this process, and as a result, they are at risk of having security defects.

In this paper, the vulnerabilities of HWMP frames are examined [15,17,18] and security requirements for HWMP are developed based on these vulnerabilities. Next, the security requirements are used to analyze the effectiveness of existing security protocols, including the BIP [8], CCMP [19], SHWMP, IBC-HWMP, ECDSA-HWMP [20], and Watchdog-HWMP [21]. It is useful to analyze these security protocols because CCMP and BIP are the default security protocols used to protect Wi-Fi management frames. SHWMP, IBC-HWMP, ECDSA-HWMP, and Watchdog-HWMP, in contrast, have been developed in recent years to protect the HWMP frames. We present a quantitative complexity comparison among the protocols and an example of security scheme for HWMP to demonstrate how the results of our research can be utilized. We hope that the application of our proposed security requirements and analyses assists future research on HWMP security.

## 2. Background

This section presents a brief explanation of the IEEE 802.11s-based WMN. The working principle behind the default wireless routing protocol, HWMP, is explained, and the concept behind its default metric, Airtime Link Metric (ALM), is reviewed. The advantages of implementing WSN with the IEEE802.11s-based WMN are also discussed.

### 2.1. IEEE 802.11s-Based Wireless Mesh Network

**Figure 1.**
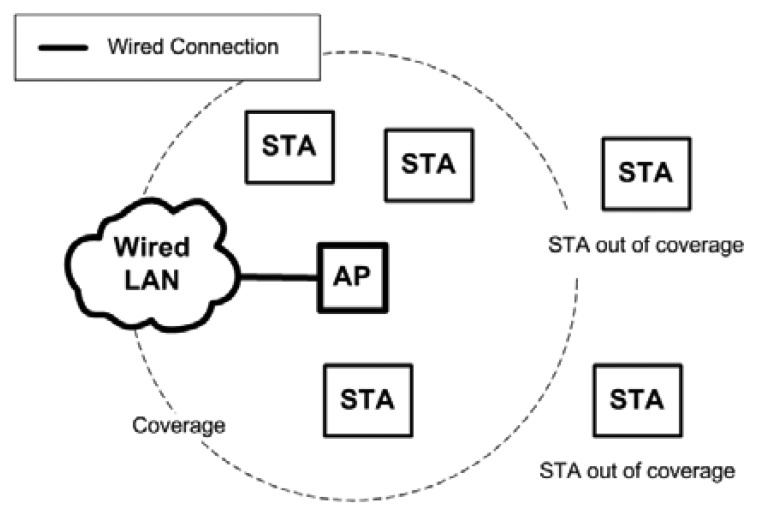
A basic wireless local area network (WLAN).

A Wireless Local Area Network (WLAN) is commonly used to provide network access to wireless device users. The most common WLAN uses access points (AP) to provide Internet connectivity to users (STAs). Each AP is connected to a wired LAN. Figure 1 shows the common way to deploy a WLAN. The coverage is limited by the range of the AP. STAs outside the coverage range cannot connect to the AP.

**Figure 2.**
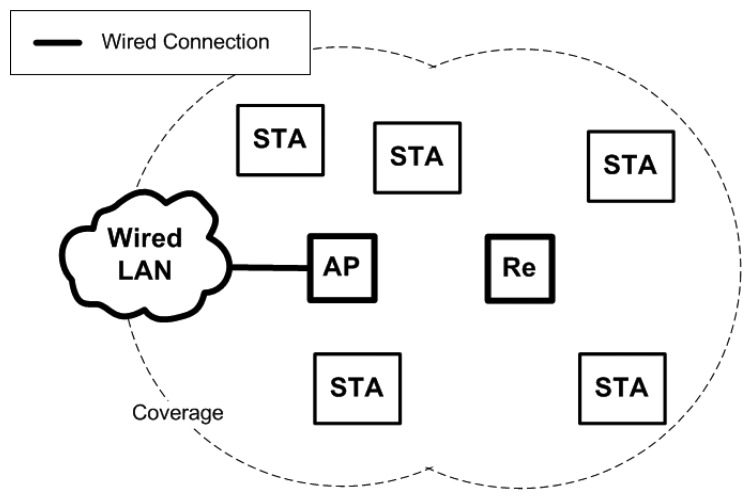
WLAN extended with a wireless repeater.

There are times when wireless repeaters are used to extend the reach of the wireless connection. Wireless repeaters are able to relay wireless frames between an AP and an STA. Wireless repeaters do not require a wired connection, but they must be connected wirelessly to an AP at all times to function. The advantage of using wireless repeaters is that a wired connection is not required. The use of wireless repeaters enables the cost of extending the wireless connection to be relatively lower, and they can be set up in areas where a wired connection cannot reach. However, the wireless connection between the AP and wireless repeater must be set statically during the set-up time. Figure 2 shows how the wireless repeater can extend the coverage of an AP to provide connection to STAs that are farther away. In the figure, the wireless repeater is denoted as Re.

**Figure 3.**
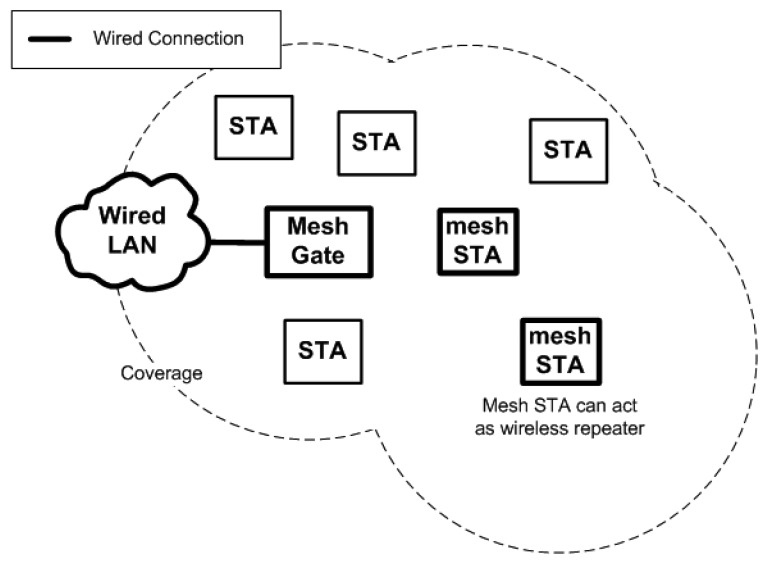
WLAN with a mesh STA.

The WMN enables STAs to provide the same functionality as an AP and repeaters. IEEE 802.11s draft defined STAs that support the mesh functionality as mesh station (mesh STA). The STAs shall be able to forward wireless frames and act as APs to connect legacy IEEE 802.11 STAs. A mesh STA that connects the WMN to the Ethernet is defined as a mesh gateway. Figure 3 shows how the wireless network works when IEEE 802.11s is implemented. The coverage will be further extended whenever a mesh STA joins the WMN.

**Figure 4.**
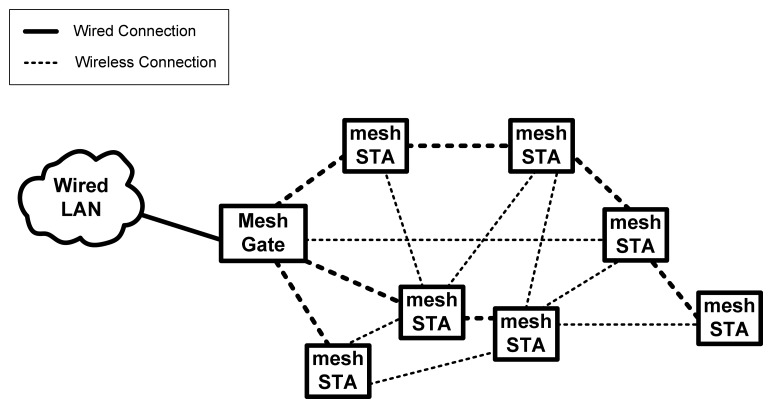
Wireless mesh network.

Figure 4 shows a WMN with additional mesh STAs. The largest difference between a repeater and a mesh STA is the latter's ability to form a wireless connection automatically and efficiently. The best path can be found by using HWMP, which is based on the shortest path algorithm. With HWMP, wireless traffic can be routed in an efficient way through path formation. ALM is used to calculate the link qualities, which are also known as the path distances. If any mesh STA is down, a new path can be formed automatically and efficiently based on HWMP.

This WMN deployment guarantees the coverage of the WLAN. However, note that the wireless connection is much slower compared to the wired connection. Thus, this WMN provides the same or larger coverage, but the connections will be slower compared to the conventional approach of using wired APs. Furthermore, multi-hop actions will cause the connection speed to be further decreased. One of the primary considerations when deploying a WMN is to make sure that all mesh STAs can reach the mesh gateway at all times with a sufficient bandwidth. The mesh gateway is also known as a gateway in WSN. In IEEE 802.11s, the mesh STA and the gateway are called a mesh point (MP) and a mesh portal (MPP), respectively.

### 2.2. Hybrid Wireless Mesh Protocol

HWMP is the default routing protocol for the IEEE 802.11s-based WMN. This protocol enables paths to be set up automatically. Paths are selected by choosing the best path based on the metrics. The concept of finding the best path is based on the Bellman-Ford or Dijkstra's algorithm [22], which solves the shortest path problem.

#### 2.2.1. HWMP Frame Formats

**Figure 5.**
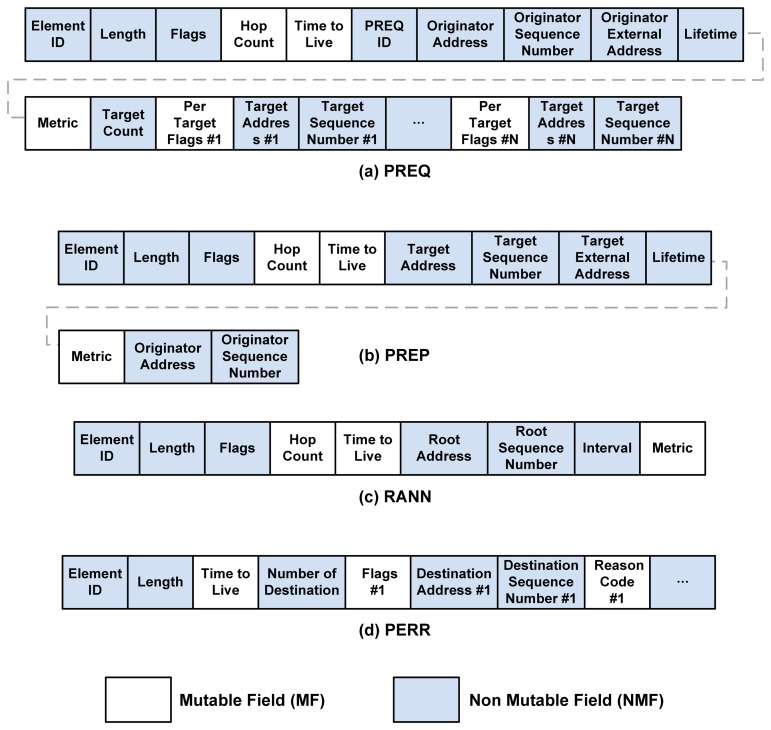
MF and NMF of PREQ, PREP, RANN, and PERR.

There are four frames directly involved in the path discovery process, including the Path Request (PREQ), Path Reply (PREP), Path Error (PERR), and Root Announcement (RANN). The frame formats are shown in Figure 5.

When considering security for routing frames, it is convenient to separate the fields into MFs and NMFs. In Figure 5, the MFs are the highlighted fields, while the rest of the frames are NMFs. MFs contain information that will be updated whenever the frames are propagated (e.g., the path metric and hop count). NMFs, in contrast, contain information that cannot be modified by an intermediate mesh STA. Note that all frames are encapsulated with the wireless MAC header (HDR) before transmissions. The HDR contains information such as the transmitter address and the receiver address. The HDR is not shown in Figure 5.

#### 2.2.2. Operation Mode [23]

IEEE 802.11s defines HWMP as a basic routing protocol for a WMN. HWMP is a hybrid routing protocol that combines a reactive mode and a proactive mode. The protocol's reactive mode operation is based on AODV, while the proactive mode uses tree-based routing. HWMP is located on layer 2; therefore, it uses MAC addresses instead of IP addresses to route message communications. In addition, note that the term ‘path selection’ is used instead of ‘routing’ in layer 2 routing. The main purpose of the on-demand routing protocol is to support the mobile mesh points, while the proactive routing protocol supports the fixed nodes. The airtime metric is a default routing metric that is used to measure the link quality. In HWMP, on-demand routing and proactive routing can simultaneously operate.

**Figure 6.**
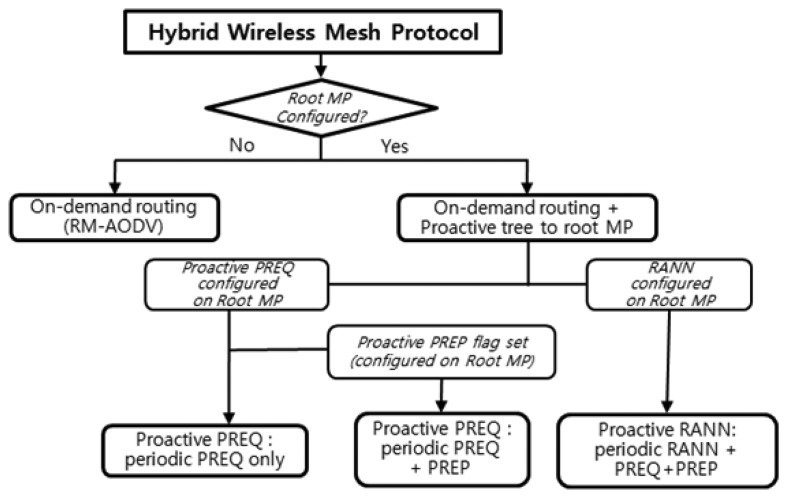
Configuration cases for HWMP [23].

HWMP sets up an MP as a root MP to build the proactive tree. Three different methods for proactive tree-building in HWMP are shown in Figure 6. The first method only uses the PREQ mechanism. The second method uses the PREQ and PREP mechanisms together. The third method uses the RANN, PREQ, and PREP mechanisms. If a MP is configured as a root MP, at least one of the proactive PREQ and RANN mechanisms should be configured. In the proactive PREQ mechanism, the proactive PREP flag can be set or not. When the proactive PREP flag is not set, then only the proactive PREQ mechanism is used to build the proactive tree.

If a root MP is configured to use the proactive PREQ mechanism, then it will periodically broadcast a proactive PREQ with an increasing sequence number. Any intermediate MPs that receive a proactive PREQ will process it in similar way as the PREQ mechanism performs on-demand path discovery.

The proactive PREP flag in the PREQ controls whether the PREP is sent in response to a proactive PREQ. If the flag is not set, then no PREP is sent in response to the reception of a proactive PREQ. This situation is called the non-registration mode. In this mode, a path tree from all of the MPs to the announced root MP is established, but the MPs are not registered proactively at the root MP. If a source MP attempts to establish a bidirectional communication path with the root MP, the source MP can send a gratuitous PREP before the first data frame to register its address with the root MP. The non-registration mode creates a path that does not strain the network and thus maintains proactive paths to the root MP.

If the proactive PREP flag is set, then the MP must send the PREP in response to the reception of a proactive PREQ. This situation is called the registration mode. In this case, MPs register with the root MP by sending a PREP in response to the proactive PREQ.

If a root MP is configured to use the proactive RANN mechanism, then it will periodically broadcast a RANN with an increasing sequence number. The RANN mechanism propagates only the path metrics to the root MP and all of the MPs in the mesh network. The RANN mechanism does not create or update any paths in the routing table. If an MP wants to create or update a path to the root MP, then it will send a unicast PREQ to the root MP, and the root MP will respond with a PREP. PREQ/PREP processing is performed in a similar manner as PREQ/PREP processing during on-demand path discovery. This mechanism establishes a forwarding tree for each MP toward the root MP. Multiple root MPs can be configured in a mesh network that is running HWMP, which means that multiple proactive trees can be built simultaneously by the different root MPs.

The hybrid routing event occurs when a root MP is configured as a registration mode. When a source MP wants to send data to a destination MP but has no path to the destination in its routing table, the source can send the data frames to the root MP. Because the mesh network is in registration mode, the root MP knows that the destination is inside of the mesh network. The root MP forwards the data frame to the destination together with an indication that both the source and destination are in the same mesh. This data frame activates the destination MP to initiate a path discovery for the destination. This procedure will establish the optimal path between the source and destination MPs, and the subsequent data frames will be forwarded on this path.

##### Path Discovery

**Figure 7.**
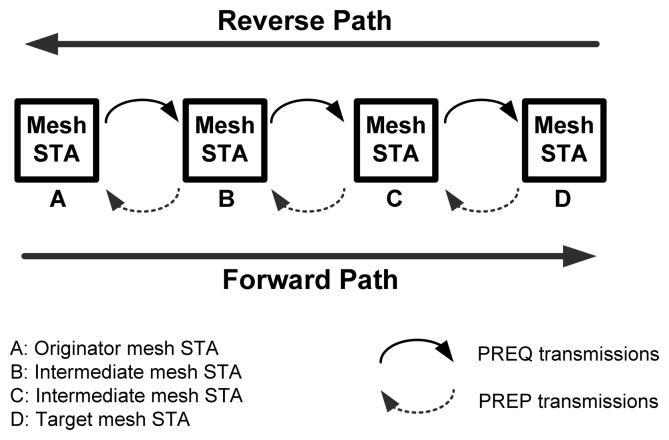
Forward and reverse path formation.

The interesting part about HWMP is how the protocol discovers paths for mesh STAs or, in other words, the wireless routing. Figure 7 shows an example of how a bi-directional path can be formed using the PREQ and PREP frames. In this example, mesh STA A is the originator, while mesh STA D is the target. Tables 1 and 2 are generated based on the scenario depicted in Figure 7. Table 1 is an example of a routing table for mesh STA C after it received the PREQ frame that originated from mesh STA A. Table 2 is an example of a routing table for mesh STA B when it receives the PREP frame replied by mesh STA D. The path discovery process is completed once the reverse and forward paths are formed. At that point, mesh STA A and D can communicate with each other using the path [A,B,C,D].

When a PREQ frame is received, the forwarding information for the originator mesh STA and the transmitter mesh STA can be updated. When a PREP frame is received, the forwarding information for the target mesh STA and the transmitter mesh STA can be updated. The forwarding information is created and updated according to the message and type of node, as described in Table 3. The table is sourced from the 802.11s drafted standards [3].

##### Forwarding Information

A HWMP sequence number is required to differentiate between the old and the new PREQ/PREP forwarding information. The sequence number is increased if a new PREQ frame is transmitted so that the other mesh STA can identify it as a new path request. The mesh STA will only process the PREQ frame if its sequence number is higher than the sequence number that it has saved in its routing table. If the sequence number is similar, the mesh STA will compare the path metric information and update the forwarding information if and only if the PREQ path metric is better. According to the drafted standards [1], the sequence number should not be increased too rapidly to avoid changing paths too often.

ALM is the default metric used for IEEE802.11s. The ALM path metric is determined by cumulating the link metric value along a path. A path with a lower path metric indicates that it is a better path (*i.e.*, it indicates a lower airtime cost or a lower transmission time). This metric measurement will influence the accuracy of the path quality measurement. If the measurement is accurate, efficient paths can be selected so that an optimum network performance can be achieved.

The hop count refers to how many transmissions are required before a frame can reach its destination. Based on the drafted standards [3], if a direct transmission occurs without hopping, the hop count field will be recorded as 1. For HWMP, there is no need for a mesh STA to store information about all intermediate mesh STAs within a path. The hop count already reflects the number of mesh STAs in the path.

Even though not all of the intermediate mesh STAs are known, the next hop mesh STA address must be identified. By referring to the routing table, a mesh STA can forward frames to the next hop address. An example of this operation is described in Figure 6 and Table 2. If mesh STA D attempts to send a packet to mesh STA A, it will transmit the packet to mesh STA C. Referring to its routing table, mesh STA C determines that the next hop for mesh STA A is mesh STA B; thus, mesh STA C can forward the packet to mesh STA B. The frame constructed by mesh STA C at this point will have transmitter address MAC C, source address MAC D, receiver address MAC B, and destination address MAC A. The forwarding information in the routing table will eventually time out. The validity period of the forwarding information is called the lifetime.

Any mesh STA can initiate the path discovery process to any mesh STA in the WMN. Once the path discovery process is complete, the best bi-directional path will be selected and the two mesh STAs can start to communicate with each other using the path. All mesh STAs can participate in the routing process. As discussed earlier, the WMN improves the coverage of a WLAN, while multi-hopping improves the throughput of a transmission, depending on the range of each mesh STA along the path.

##### Airtime Link Metric (ALM)

ALM is used as the default routing metric for IEEE 802.11s. Periodically, a test frame will be sent from a mesh STA to its neighbor mesh STA through a link. The test frame error rate (e.g., the ratio of the failed transmissions to the total transmissions) is calculated over time. Based on the data rate of the link and the frame error rate, the ALM can be calculated as:

(1) $$C_{a}=[O+\frac{B_{t}}{r}]\frac{1}{1-e_{f}}$$

where *O* is the overhead caused by the physical layer (PHY); its size varies depending on PHY. *B_t_* is the size of the test frame. Both *O* and *B_t_* are constant values. *r* is the data rate, and *e_f_* is the frame error rate. Airtime cost is denoted by *C_a_*. With these variables, the estimated transmission time over a link can be calculated. This value is used as the metric for the remainder of this paper.

Note that ALM will be used to calculate the metric of the links, but it is not directly involved in the path formation process. HWMP is the routing protocol that controls the path formation process. By using ALM, the best path can be found based on the expected transmission time and error rate, rather than the hop count. If a large number of mesh STAs are placed relatively near each other in an 802.11s-based WMN, the performance of the network has a high probability of increasing.

ALM takes both the transmission time and error rate parameters into consideration when computing the link quality (*i.e.*, the link metric). Therefore, if HWMP utilizes ALM as the metric, this protocol will be able to route wireless frames more efficiently compared to the hop count-based metric. Of course, scholars have developed a variety of metrics, each with its own capabilities, and these may outperform the default ALM [24]. Examples of these capabilities include load effect sensing, interference reduction, and channel allocation. Each of these metrics considers other parameters in its equation, but the core parameters are still the transmission time and the error rate.

##### Wireless Sensor Network (WSN)

WSN have been implemented in a variety of ways, but the architecture is quite similar across implementations. For example, WirelessHART was one of the first open standards available for use in the process control field. The architecture for a WirelessHART network is defined in its data sheet [25]. WirelessHART consists of three main elements, namely field devices, gateways, and network managers. The field devices form a mesh network, while all of these devices are then connected to the gateway. The gateway connects the mesh network to the network manager and the other servers. We are interested in the mesh network (or mesh nodes) and the gateway. The gateway is the only means for data to be sent in and out of the mesh network.

##### The IEEE 802.11s-Based WMN for WSN

Wi-Fi technology (or IEEE 802.11) is known to exhibit higher bandwidth compared to Bluetooth or ZigBee [6]. Because IEEE 802.11s is a data link layer implementation, it uses the same PHY layer as common Wi-Fi devices, such as 802.11a/b/g/n. Therefore, the IEEE 802.11s standard will also exhibit a high bandwidth capacity. This capacity means that it can support high levels of traffic and a large number of mesh STAs. This high level of traffic support is appropriate for certain WSN applications that require a large bandwidth. In fact, Wi-Fi technology has already been used for WSN applications, such as privacy-preserving childcare and safety services [26], as well as in sensor systems for the analysis of highly dynamic movement techniques [27].

The 802.11s standard is compatible with other non-mesh Wi-Fi devices. Normal STAs with mesh functionality can still connect to the WMN by acting as an AP and connecting with the mesh STA. This compatibility means that WSN that utilize the IEEE 802.11s-based WMN will be able to integrate with existing Wi-Fi networks easily. The integration of several wireless systems into the same wireless network is also possible.

Iera *et al.* [28] studied the use of smart phones as a mesh router or gateway. Their results indicated that it is technically possible for 802.11 devices to be converted into mesh STAs through layer-2 implementation (*i.e.*, there is no need to modify the device hardware). If the mesh STA is simultaneously connected to the wired LAN, it can be used as a gateway. This connection allows the WSN to be deployed faster because the existing network infrastructure can be utilized. In addition, fewer new network devices are required. Many Wi-Fi devices will likely be converted into mesh STAs to assist in the WMN routing process. Therefore, the effect of having additional mesh STAs on network performance should be investigated.

## 3. Attacks on HWMP

Attacks on the routing protocol can be divided into external and internal attacks. External attacks refer to attacks launched by an attacker that does not have access to the network. Internal attacks refer to attacks launched by an authenticated mesh STA within the network.

### 3.1. External Attacks

#### 3.1.1. Flooding Attacks

A flooding attack can deplete network resources [10,17]. An attacker can broadcast PREQ frames continuously to flood the WMN. PREQ frames are continuously propagated throughout the WMN. An attacker can use target mesh STA addresses that do not exist in the WMN to ensure that the PREQ frames will be propagated many times (or at least until they are Time-to-Live timed out) [10]. Eventually, network resources will be used up to process the PREQ frames.

The attacker can also continuously broadcast RANN frames. This continuous broadcasting will force other mesh STAs to respond to the RANN by requesting a path to reach the root mesh STA. The number of path requests depends on the number of mesh STAs. If the attacker continues to broadcasting RANN with different root mesh STA addresses, the network will be flooded with HWMP frames very quickly.

If the paths are known, an attacker can send out fake PERR frames to produce a false error report [17]. This false error report will result in mesh STAs deleting the routing information for the target mesh STA in their routing table. Mesh STAs will be forced to request paths again because of the fake PERR frames. In the end, the network will be flooded with HWMP frames.

#### 3.1.2. Path Diversion Attacks

An attacker can increase the originator sequence number of a PREQ frame to trick other mesh STAs into processing the forwarding information as new information. In this case, the routing table for each mesh STA will be updated based on the assumption that the PREQ frame contains new information. The attacker can also lower the path metric value to trick other mesh STAs into believing that a path is a better one when, in fact, it is inferior to the previous paths.

**Figure 8.**
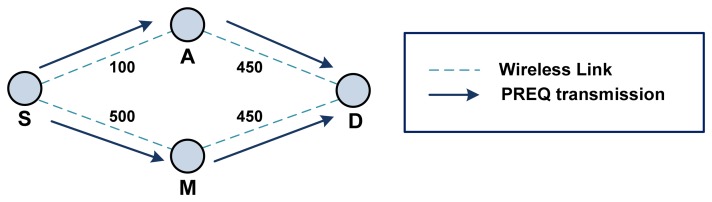
Example of a path diversion attack.

Information from the transmitted frames is processed according to Figure 8 as follows:

##### Case 1: Fake Path Metric

S: S → Broadcast: PREQ [S - D] [Metric = 0]
A: A → Broadcast: PREQ [S - D] [Metric = 100]
M: M → Broadcast: PREQ [S - D] [Metric = 10]

Note that S: S → Broadcast: PREQ [S - D] [Metric = 0] indicates that the sender of the frame is S; the frame is broadcasted; the type of frame is PREQ; the source of the frame is S; the destination is D; and the current metric is 0.

D calculates the metric of path {S↔A↔D} to be 550, while {S↔M↔D} is calculated to be 460 because of the fake metric field. Therefore, path {S↔M↔D} will be chosen as the better path, and D will thus transmit the PREP frame to M.

##### Case 2: Fake Originator Sequence Number

S: S → Broadcast: PREQ [S - D] [Metric=0] [Originator Sequence Number = 100]
A: A → Broadcast: PREQ [S - D] [Metric=100] [Originator Sequence Number = 100]
M: M → Broadcast: PREQ [S - D] [Metric=500] [Originator Sequence Number = 101]

This time, D knows that the path {S↔A↔D} has a lower path metric, but D determines the path {S↔M↔D} to be a new path and thus chooses the new path. D will thus transmit the PREP frame to M.

Figure 8 shows an example of a path diversion attack. Path {S↔A↔D} is the better path with a metric of 550, while the metric for {S↔M↔D} is 950. However, the malicious mesh STA M can divert a path to go through the malicious mesh STA itself by reporting a higher sequence number or a lower metric value.

#### 3.1.3. Wormhole and Blackhole Attacks

A wormhole is created so that an attacker can spy on data packets [17,29]. The attacker can first use a path diversion attack to ensure that the path formed will go through itself. A blackhole is created so that data packets can be dropped by the attacker for forwarding [17,26,30]. Again, the attacker must first use a path diversion attack to ensure that the formed path will go through itself. Once the path is formed, the attacker can start dropping data packets instead of forwarding them. Note that wormhole and blackhole attacks actually target the data packets instead of data link layer's frames. However, these attacks are performed by exploiting HWMP to form paths that move through the attacker, as per the path diversion attacks.

#### 3.1.4. Impersonation Attacks (Loop)

**Figure 9.**
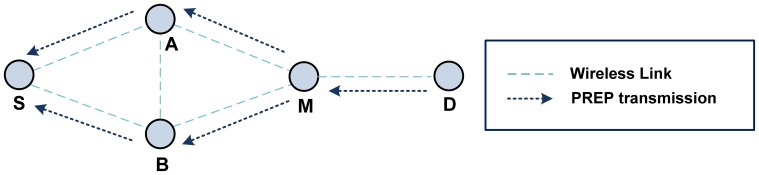
Example of an impersonation attack (loop).

There are four address fields in the HWMP frames: transmitter, receiver, originator, and target. An attacker can impersonate other mesh STAs by changing the addresses in these fields. The impersonation attack can be used to create a loop [29] by manipulating the transmitter and receiver address fields. Figure 9 shows an example of a looping attack.

S initiates a PREQ frame (as usual) until the PREQ frame reaches D. D will then reply to the request with a PREP frame. The malicious mesh STA M will impersonate A and B to confuse these STAs. A loop between A and B will be formed. The sequence of transmitted frames used by STA M to create a loop is as follows:

D: D → M: PREP [S - D]
M: B → A: PREP [S - D]
M: A → B: PREP [S - D]
A: A → S: PREP [S - D]
B: B → S: PREP [S - D]

When S receives the PREP frame, it will choose either A or B as the next hop. At this point, A receives information that the next hop to reach D is B, while B receives information that the next hop to reach D is A. When S sends a data packet to D, it will transmit the packet to either A or B. Then, A and B will forward the packets to each other continuously. As a result, the data packet will never reach D *via* M.

#### 3.1.5. Impersonation Attacks (Path Diversion)

**Figure 10.**
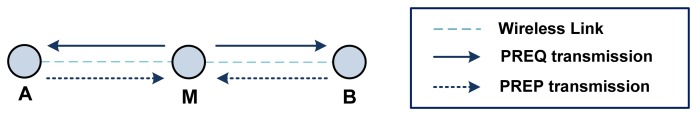
Example of an impersonation attack (path diversion).

It is possible for an attacker to impersonate another mesh STA to initiate the path request. This impersonation is performed by manipulating the originator and target address fields. Figure 10 shows an example of how an impersonation attack can be used with a path diversion attack.

Information about the transmitted frames is processed according to Figure 10 as follows:

M: M → Broadcast: PREQ [A - B] [Metric = 10]
M: M → Broadcast: PREQ [B - A] [Metric = 10]
A: A → M: PREP [A - B] [Metric = 0]
B: B → M: PREP [B - A] [Metric = 0]

First, a malicious mesh STA M will broadcast a PREQ frame with the originator address set to B, the target address set to A, and the metric set to 10. Then, it will broadcast another PREQ frame with the originator address set to A, the target address set to B, and the metric set to 10. Both A and B will reply to the request to form the path {A↔M↔B}.

#### 3.1.6. Replay Attack [31]

To launch a replay attack, the attacker eavesdrops on some communication sessions and saves the routing messages, and later replays these messages by spoofing the communicating parties. By sending old RPEQ messages and spoofing the MAC address of the victim, the attacker convinces the destination node into believing that the victim node is trying again to communicate with the destination node. The destination node replies with PREP for the attacking node. At this point, the attacker starts communication with the destination node while the destination node believes the attacker is the original source node S. This attack scenario is possible when the victim node reboots.

#### 3.1.7. Passive Attacks (Eavesdropping)

HWMP frames contain routing information for the WMN. Routing information can be obtained by sniffing the HWMP frame exchanges. The information can be useful or useless, depending on the application of the WMN. In some cases, the number of mesh STAs within a WMN should be kept a secret. If the WMN is used in a military field, the enemy might be able analyze military strength based on the number of mesh STAs. In addition, metrics and routing path information can be used to analyze the position and distance of other mesh STAs [17]. Finally, routing information can be used by attackers to easily launch active attacks [32].

The easiest way for an attacker to gain this type of information is by pretending to be a root mesh STA and broadcasting the RANN. Upon receiving the RANN, all mesh STAs will respond by sending out PREQ frames with the root mesh STA address as the target. Once the attacker receives the PREQ frames from all mesh STAs, the attacker will know the path metrics, the number of mesh STAs, and the MAC addresses of all mesh STAs within the WMN.

### 3.2. Internal Attacks

Internal attackers are users authorized to join the WMN. Internal attackers have all of the required security keys. Link-based security measures are unable to prevent internal attacks. The goals of internal attacks are different than external attacks. Most likely, internal attackers will not flood the WMN because they are also legitimate users of the network. Moreover, they most likely have different reasons for attacking the network compared to external attackers.

Greedy users will try to deny the access of other users to obtain a better share of the network's bandwidth [33]. This denial of access can be performed by creating a blackhole that sinks the data packets of the other mesh STAs. This type of attack will prevent other users from using the network, but it will not disrupt the operation of the network. In the end, the attacker will be able to monopolize all of the network's bandwidth resources.

Another type of internal attack involves creating a wormhole to spy on data packets [33]. For example, a company staff member might be interested in his/her company's private secrets. The wormhole can be used to collect data packets to obtain private information. The network operation will not be disrupted, but the attacker will be able to collect the information that he/she wants.

As discussed earlier, blackholes and wormholes attack data packets instead of the data link layer's frames. The blackhole or the wormhole can be created by exploiting HWMP with a path diversion attack. Path metrics, sequence numbers, originator addresses, and target addresses can all be manipulated to enable the path diversion attack. Importantly, the security requirements to prevent external *versus* internal attacks are different from each other because internal attackers have the required security keys to authenticate the frames.

From the insider attack on AODV [15], we can easily identify the vulnerabilities of the reactive mode HWMP frames and PREQ frames by the destination node in hybrid mode HWMP. Tables 4 and 6 list the message misuses and achievable misuse goals for PREQ, PREP, and PERR, respectively. Most of attack activities are on NMFs [15]. Therefore, the integrity and source origination authentication of the NMFs must be provided.

## 4. HWMP Security

### 4.1. Security against External Attacks

The main goal in protecting the WMN from external attacks is to keep attackers out of the network. To prevent such attacks, 802.11s has defined the Simultaneous Authentication of Equals (SAE) and 802.1x authentication protocols. The outputs of the authentication protocols are authenticated keys, such as the Pairwise Transient Key/Group Temporal Key (PTK/GTK). These keys can be used to authenticate frames. Non-authenticated frames will not be serviced by other mesh STAs. Note that PTK/GTK is only used for a point-to-point link protection. Most of the external attacks can be protected with data-origin authentication and data integrity service. Hereafter we call “data-origin authentication” and “data integrity” service as “authentication and integrity” service.

A message authentication code (*MAC* not to be confused with MAC which stands for medium access control) or a digital signature scheme can provide integrity and authentication service [34]. *MAC* is one-way hash function with key dependency. *MAC* is generated just like a one-way hash function but a symmetric key is also included. Only an entity with the same symmetric key can verify the *MAC*. So the verifier can get integrity and authentication service. Digital signature schemes with public-key cryptography and one-way hash function is also a good tool to authenticate the signer and the integrity of the signed message. The message is hashed first and a signer encrypts the hash with her/his private key, thereby signing the message. Because the signer's public key is made public, any entity with the message and the corresponding signature can verify the signature.

Suppose that an unauthenticated frame had been transmitted and subsequently captured by an attacker. The attacker can then modify the contents of the frame and retransmit it. This modified frame will be considered as a legal frame by a receiving node or a destination node because it is not able to detect the modification. If *MAC* or digital signature were attached to the frame, this type of attack can be protected.

Confidentiality is another important security measure that protects the privacy of frames. Confidentiality is ensured by encrypting frames with an algorithm, such as RC4 or the Advanced Encryption Standard (AES) [35]. RC4 is a typical stream encryption algorithm and AES is a typical block encryption algorithm. After encryption, external attackers will not be able to read the transmitted frames.

Table 7 lays out the external attacks, the required security services to protect them, and the relevant HWMP frames and fields involved. Note that only link-to-link based security services are required here. The security services required to prevent external attacks are quite simple and straightforward; the more interesting topic in WMN security is preventing internal attacks.

Because an attacker cannot capture the correct contents of an encrypted message without knowing the key, encryption protects a passive attack. Digital signature or *MAC* is a good tool to authenticate the communicating messages. A non-authenticated broadcasting message would not be delivered by the intermediate nodes along the network and thus the flooding attack will be protected. A well designed authentication protocol with the use of freshness identifiers such as nonce or time-stamp can prevent the replay attack. Any replay of old messages violates the freshness of the authentication protocol [36]. Because path diversion attack and impersonation attack in HWMP are launched by modifying some information in a message, the message integrity check service can protect these types of attack. Especially end-to-end integrity service is required for the NMFs because NMFs should not be changed from the source to the destination along the transmission path.

### 4.2. Security against Internal Attacks

The real problem with the WMN is the possibility of an internal attack. Internal attackers can exploit HWMP by using a path diversion attack to create a blackhole or wormhole. An internal attacker may also pretend to be a root mesh STA. When a mesh STA tries to send packets to a target, it will initialize the path discovery process. Before the path is found, it will first use the root mesh STA to forward its packet. This way, it will be possible for the attacker to spy on the first few data packets.

Table 8 describes the internal attacks, the required security services to protect them, and the relevant HWMP frames and fields involved. By lowering the metric to an extraordinary small value compared to that of the other competing paths, the attacker can divert the route to meet the needs of the attacker. By illegally increasing the sequence number of the routing frame, the attacker can cause the victim nodes to treat this routing message frame as the most recent one. To protect against a path diversion attack, a scheme to detect a fake path metric is required. End-to-end authentication and integrity services are required for the NMF to prevent an intermediate mesh STA from modifying the sequence number. As described in Section 2.2.1, an attacker can set itself up as a root node with RANN and PREQ. To prevent this type of attack, a permission level should be implemented so that only authorized mesh STAs can declare themselves as a root mesh STA. By changing the originator and target address fields, an attacker can impersonate other nodes. End-to-end authentication and integrity services are required for the NMF including the HDR field to prevent an intermediate mesh STA from impersonating another mesh STA.

### 4.3. HWMP Security Requirements

Table 9 describes the security service requirements and the relevant message fields involved to protect HWMP message from possible internal and external attacks. The requirements are determined based on the information from Tables 7 and 8.

The authentication and integrity services (point-to-point) are required for all frames and fields. If those services are in use, a receiver can verify the origin of the frame. The receiver will also be able to verify that the frame has not been modified by attackers. This security service is sufficient to protect HWMP frames from all external attacks except eavesdropping.

An encryption service can only be provided for the MF and NMF. Frames must be encrypted in a point-to-point manner instead of an end-to-end manner because intermediate mesh STAs must read the frame information as well. Encryption should only be used if the application requires high secrecy because encryption is computationally expensive. Network administrators should be given the option to disable encryption.

Authentication and integrity (end-to-end) services are required only for the NMF. These services prevent intermediate mesh STAs from modifying the information in this field. With these services implemented, intermediate mesh STAs will not be able to modify the HWMP sequence number or to impersonate the originator/target mesh STAs.

A root permission level is required for the PREQ and RANN frames, depending on the proactive tree-building mode. Meanwhile, a permission level is required only for the NMF. This permission level will prevent a normal mesh STA from declaring itself as the root. For example, Identity Based Signature (IBS) schemes can be used on the NMF of the PREQ and RANN frames. The identity used in the schemes can include MAC addresses and the root permission as the input. With a permission level implemented, all mesh STAs will be able to determine whether the mesh STA is a true root or not. Of course, using this type of scheme requires other considerations, such as key distribution methods and computational overhead, but implementing the root permission level provides one possible security service.

Internal attackers have the required security keys to authenticate the HWMP frames in a point-to-point manner; thus, they can easily forge the path metric. End-to-end-based integrity services will not be able to stop internal attackers from forging path metrics because the MF will be updated whenever the HWMP frames are propagated. Therefore, an efficient forgery detection method is required to prevent intermediate mesh STAs from transmitting HWMP frames with fake path metrics.

For example, mesh STAs can check the frames propagated by their neighbors. When a mesh STA transmits an HWMP frame, it should check the frames propagated by its neighbors by comparing them with the frame it transmitted. The path metric in the frames propagated by its neighbors should not be less than the path metric of the original frame. Of course, this is just one example of how the forgery detection method can be implemented; there will be many factors to consider in designing a real-life forgery detection method, such as the computational overhead and the storage resources. IBC-HWMP provides one way of implementing this type of forgery detection method, and the working principle of this detection method is explained in the next section.

In short, Table 9 can be divided into two parts. The first two columns, authentication and integrity (point-to-point) and encryption (point-to-point), are required to protect the HWMP frames against external attacks. The last three columns, authentication and integrity (end-to-end), root permission level, and forgery detection method, are required to protect the HWMP frames against internal attacks. A security protocol can protect HWMP against both external and internal attacks if it can satisfy all of the requirements mentioned in the table.

Existing authentication protocols in 802.11s provide the necessary link-based security key, such as PTK/GTK. In other words, point-to-point-based security services for HWMP frames are easily implemented. The difficult part involves the security service requirements for internal attacks. Future development of HWMP security protocols should therefore concentrate on security service requirements for internal attacks.

## 5. Analysis of HWMP Security Protocols

### 5. 1 CCMP and BIP

Assuming that HWMP frames are treated as normal management frames, they can be protected according to the 802.11w confidentiality and integrity protocols. All of the HWMP frames (including the MF and NMF) will be treated as a frame body, and they will be encapsulated with a MAC HDR before transmission. Detailed information on CCMP and BIP can be found in the standards [8,19].

**Figure 11.**
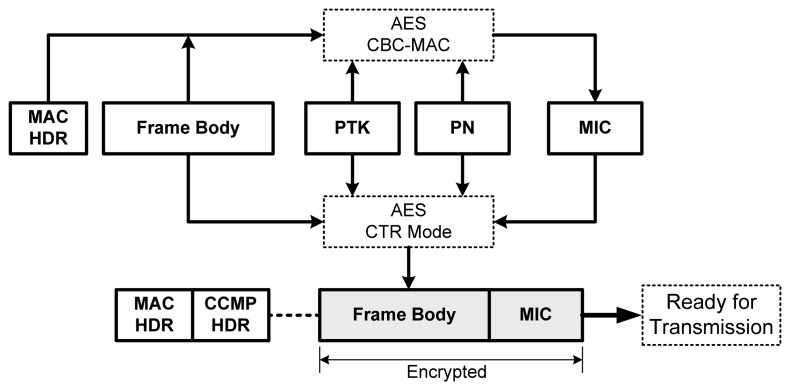
The working principle of CCMP.

Figure 11 explains how frames can be protected using CCMP. Only the frame body and the message integrity code (MIC) will be encrypted. CCMP HDR is the header that contains the information required for decryption and integrity checks, such as the packet number (PN), Initialization Vector (IV), and key ID. MAC HDR contains information such as the transmitter and receiver MAC addresses.

**Figure 12.**
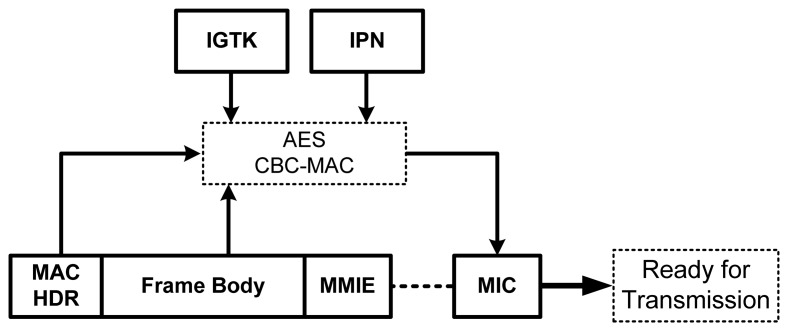
The working principle of BIP.

BIP introduces the Integrity GTK (IGTK) and the integrity packet number (IPN), which are solely used in BIP. The use of IGTK and IPN is similar to the use of PTK and PN in CCMP. Figure 12 shows how the frames can be protected using BIP. The management MIC Integrity Element (MMIE) contains the information required for the integrity check, including the IPN and the key ID.

Table 10 lists the security services provided by CCMP and BIP. The only difference between them is confidentiality; BIP does not provide encryption. BIP is intended for broadcast frames, while CCMP is intended for unicast frames. However, the use of the protocols in this security context is interchangeable. We generalize the security services provided by both security protocols in Table 10, and we highlight the security services that are not addressed.

### 5.2. Secure Hybrid Wireless Mesh Protocol (SHWMP)

Islam *et al.* [10] proposed SHWMP to secure HWMP frames from external attacks. The Merkle tree algorithm is used to generate the MIC for MF. This MIC is then encrypted with PTK/GTK to provide authentication and integrity services for the MF. The NMF is encrypted using the PTK/GTK, which also protects confidentiality. For the PREQ message, the hop count, time to live (TTL), metric, and per destination flag are mutable elements. For the PREP and RANN frames, the hop count, TTL, and metric are mutable elements. SHWMP does not provide any security service for PERR frames.

#### 5.2.1. A Secure Scheme for Reactive Routing

Let us assume a network topology scenario with a source MP S that can reach destination MP D in three hops. Here, we define the number of hops as the number of links on a path. There are two arbitrarily connected intermediate nodes and that may be reached after the 1st and 2nd broadcasting, respectively. Subsequently, the message reaches the destination MP D.

SHWMP runs a secure routing scheme on the network scenario as follows:

(1) $$\text{S}\to^{*}:{\text{MAC}}_{\text{GTK}}\text{root}(\text{S}),\{\{{\text{v}}_{\text{i}},\text{authplay}({\text{v}}_{\text{i}}),\ldots \}\},{\left\{\text{PREQ}-\text{MF}\right\}}_{\text{GTK}}$$

(2) $${\text{I}}_{1}\to^{*}:{\text{MAC}}_{\text{GTK}}\text{root}({\text{I}}_{1}),\{\{{\text{v}}_{\text{i}},\text{authplay}({\text{v}}_{\text{i}}),\ldots \}\},{\left\{\text{PREQ}-\text{MF}\right\}}_{\text{GTK}}$$

(3) $${\text{I}}_{2}\to^{*}:{\text{MAC}}_{\text{GTK}}\text{root}({\text{I}}_{2}),\{\{{\text{v}}_{\text{i}},\text{authplay}({\text{v}}_{\text{i}}),\ldots \}\},{\left\{\text{PREQ}-\text{MF}\right\}}_{\text{GTK}}$$

(4) $$\text{D}\to {\text{I}}_{2}:{\text{MAC}}_{\text{PTK}}^{{\text{D},\text{I}}_{2}}\text{root}(\text{D}),\{\{{\text{v}}_{\text{i}},\text{authplay}({\text{v}}_{\text{i}}),\ldots \}\},{\left\{\text{PREP}-\text{MF}\right\}}_{\text{PTK}}^{{\text{D},\text{I}}_{2}}$$

(5) $${\text{I}}_{2}\to {\text{I}}_{1}:{\text{MAC}}_{\text{PTK}}^{{\text{I}}_{2},{\text{I}}_{1}}\text{root}({\text{I}}_{2}),\{\{{\text{v}}_{\text{i}},\text{authplay}({\text{v}}_{\text{i}}),\ldots \}\},{\left\{\text{PREP}-\text{MF}\right\}}_{\text{PTK}}^{{\text{I}}_{2},{\text{I}}_{1}}$$

(6) $${\text{I}}_{1}\to \text{S}:{\text{MAC}}_{\text{PTK}}^{{\text{I}}_{1},S}\text{root}({\text{I}}_{1}),\{\{{\text{v}}_{\text{i}},\text{authplay}({\text{v}}_{\text{i}}),\ldots \}\},{\left\{\text{PREP}-\text{MF}\right\}}_{\text{PTK}}^{{\text{I}}_{1},\text{S}}$$

Note that and represent the routing information elements of the PREQ and PREP frames without the MFs, respectively. {{v_i_,authplay(v_i_),…}} denotes the set of MFs and the values required to authenticate the set over the Merkel tree. MAC_k_root(X) represents the *MAC* of the Merkel tree's root created by node S using a shared key k on the MFs of its routing protocol message. {M}_K_ denotes a cipher-text message M encrypted with a symmetric key k. ${\left\{\text{M}\right\}}_{\text{k}}^{\text{A},\text{B}}$ denotes a cipher-text message M encrypted with a symmetric key k shared between entity A and B.

#### 5.2.2. A Secure Scheme for Reactive Routing

For the given scenario in subsection 5.2.1, SHWMP runs reactive secure routing mechanisms for RANN, PREQ, and PREP as follows:

(8) $$\text{R}\to^{*}:{\text{MAC}}_{\text{GTK}}\text{root}(\text{R}),\{\{{\text{v}}_{\text{i}},\text{authplay}({\text{v}}_{\text{i}}),\ldots \}\},{\left\{\text{RANN}-\text{MF}\right\}}_{\text{GTK}}$$

(9) $${\text{I}}_{1}\to^{*}:{\text{MAC}}_{\text{GTK}}\text{root}({\text{I}}_{1}),\{\{{\text{v}}_{\text{i}},\text{authplay}({\text{v}}_{\text{i}}),\ldots \}\},{\left\{\text{RANN}-\text{MF}\right\}}_{\text{GTK}}$$

(10) $${\text{I}}_{2}\to^{*}:{\text{MAC}}_{\text{GTK}}\text{root}({\text{I}}_{2}),\{\{{\text{v}}_{\text{i}},\text{authplay}({\text{v}}_{\text{i}}),\ldots \}\},{\left\{\text{RANN}-\text{MF}\right\}}_{\text{GTK}}$$

(11) $$\text{D}\to {\text{I}}_{2}:{\text{MAC}}_{\text{PTK}}^{\text{D},{\text{I}}_{2}}\text{root}(\text{D}),\{\{{\text{v}}_{\text{i}},\text{authplay}({\text{v}}_{\text{i}}),\ldots \}\},{\left\{\text{PREQ}-\text{MF}\right\}}_{\text{PTK}}^{\text{D},{\text{I}}_{2}}$$

(12) $${\text{I}}_{2}\to {\text{I}}_{1}:{\text{MAC}}_{\text{PTK}}^{{\text{I}}_{2},{\text{I}}_{1}}\text{root}({\text{I}}_{2}),\{\{{\text{v}}_{\text{i}},\text{authplay}({\text{v}}_{\text{i}}),\ldots \}\},{\left\{\text{PREQ}-\text{MF}\right\}}_{\text{PTK}}^{{\text{I}}_{2},{\text{I}}_{1}}$$

(13) $${\text{I}}_{1}\to R:{\text{MAC}}_{\text{PTK}}^{{\text{I}}_{1},\text{R}}\text{root}({\text{I}}_{1}),\{\{{\text{v}}_{\text{i}},\text{authplay}({\text{v}}_{\text{i}}),\ldots \}\},{\left\{\text{PREQ}-\text{MF}\right\}}_{\text{PTK}}^{{\text{I}}_{1},\text{R}}$$

(14) $$\text{R}\to {\text{I}}_{1}:{\text{MAC}}_{\text{PTK}}^{\text{R},{\text{I}}_{1}}\text{root}(\text{R}),\{\{{\text{v}}_{\text{i}},\text{authplay}({\text{v}}_{\text{i}}),\ldots \}\},{\left\{\text{PREQ}-\text{MF}\right\}}_{\text{PTK}}^{\text{R},{\text{I}}_{1}}$$

(15) $${\text{I}}_{1}\to {\text{I}}_{2}:{\text{MAC}}_{\text{PTK}}^{{\text{I}}_{1},{\text{I}}_{2}}\text{root}({\text{I}}_{1}),\{\{{\text{v}}_{\text{i}},\text{authplay}({\text{v}}_{\text{i}}),\ldots \}\},{\left\{\text{PREP}-\text{MF}\right\}}_{\text{PTK}}^{{\text{I}}_{1},{\text{I}}_{2}}$$

(16) $${\text{I}}_{2}\to \text{D}:{\text{MAC}}_{\text{PTK}}^{{\text{I}}_{2},\text{D}}\text{root}({\text{I}}_{2}),\{\{{\text{v}}_{\text{i}},\text{authplay}({\text{v}}_{\text{i}}),\ldots \}\},{\left\{\text{PREP}-\text{MF}\right\}}_{\text{PTK}}^{{\text{I}}_{2},\text{D}}$$

The node S is configured as root R. In the proactive RANN mode, the root node will broadcast RANN, as described in Equation (8), which is propagated to D via the intermediate nodes and , as described in Equations (9) and (10). If D aims to create or update a path to root MP, it unicasts a PREQ frame with Equation (11). The subsequent message transmission along the path will follow Equations (12) and (13). Then, the corresponding PREP response will follow Equations (14–16).

Proactive PREQ modes, including registration and non-registration, will handle the PREQ/PREP transmission in ways similar to those explained above.

The security services provided by SHWMP are listed in Table 11. We highlight the security services that are not addressed. SHWMP concentrates on protecting HWMP frames from external attacks, but the protocol is not complete. Only the authentication and integrity of the MF is protected in point-to-point link. GTK is only valid between peering neighbors. The biggest flaw in SHWMP is that it does not provide any protection from internal attacks, nor does it provide the secure end-to-end authentication and integrity for broadcasting and unicasting messages. This protocol also does not have any security schemes for PERR HWMP routing protocol frames. This protocol does not provide the authentication and integrity assurance of NMF from the source node for the intermediate nodes on the transmission path to protect NMF modification attacks. In addition, an anti-replay scheme does not exist in this protocol.

### 5.3. IBC-HWMP, ECDSA-HWMP and Watchdog-HWMP

Ben-Othman *et al.* proposed IBC-HWMP [16] and ECDSA-HWMP [31]. The MAC address is used as the identity for all mesh STAs in the IBC-HWMP. For the two proposed schemes, a private key is used to sign the MF of PREQ and PREP such that the authentication and the integrity of the MF are protected. Public keys are distributed to verify signatures. The identity based signature scheme and ECDSA are used to generate the signature for IBC-HWMP and ECDSA-HWMP, respectively. The digital signature on the broadcasting PREQ message can cause a lot of message drops if the mesh node cannot process the signature verification at line speed. Furthermore, to implement the ECDSA-HWMP, the mesh network system must transmit the digital certificate to the signature verifier and maintain the revocation list of the nodes' certificate, which requires the third trusted certificate authority (CA). In wireless mesh network, the implementation of CA is impractical.

**Figure 13.**
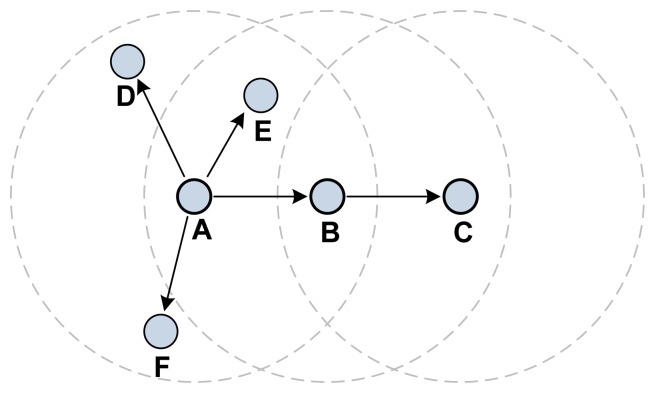
IBC-HWMP detection method.

The authors also suggested that the MF should be observed to detect illogical changes [32]. This scheme to detect illogical changes is called a Watchdog-HWMP. Figure 13 shows an example of this MF forgery detection method. The example starts with A broadcasting a PREQ frame to its neighbors. When B propagates the PREQ to C, A should capture this frame and observe the changes to the MF. When A first broadcasts the PREQ frame, D, E, and F propagate the PREQ frame as well. A must therefore capture all PREQ frames propagated by its neighbors and compare them with the original PREQ. This means that all mesh STAs must keep information on the PREQ frames they have transmitted and compare them with all PREQ frames propagated by their neighbors. Because the non-mutable field is a major target for internal attacks, illegal modification or creation in this field must be detected or prevented. Inclusion of the monitoring of message drop into watchdog processing is recommended.

The security services provided by IBC-HWMP are listed in Table 12. IBC-HWMP does not explain how to protect RANN and PERR, nor does it have end-to-end and point-to-point authentication and integrity for NMFs. We highlight the security services that are not addressed. This protocol does not provide integrity assurance of NMF from the source node for the intermediate nodes on the transmission path to protect NMF from modification attacks. In addition, no anti-replay scheme exists in this protocol.

### 5.4. The Comparison of Complexity

In terms of computation time comparison, we cannot tell the exact time of each algorithm in Table 13. It includes variety of cryptographic algorithms such as hash evaluation, symmetric encryption, operation over finite field, and operation over elliptic curve. Some algorithms such as integer multiplication over a finite field and scalar multiplication of elliptic curve might have operand dependent execution time. But we can compare the computation complexity of the algorithms. Among pairing (P), elliptic curve point exponentiation (E), a scalar multiplication of elliptic curve (M), elliptic curve point addition (A), integer multiplication over integer finite field (MI), symmetric encryption/decryption (Ec), and hashing (H), pairing has the most complex operation and hashing has the least complex operation. We can sort the computational complexity of the algorithms as follows: P > E > M > A > MI > Ec > H.

Here are some considerations which make the complexity comparison simple. CCMP and BIP are not used at the as time because BIP is only for data integrity and CCMP is for data integrity and confidentiality. MAC computation time in SHWMP was approximated to hash evaluation time. For the computation complexity of SHWMP, refer to [10]. Because IBC-HWMP requires paring operations much heavier than hashing, we do not take hash evaluation into account. Pre-computation (offline computation) time is also not taken into account. Hash evaluation and off-line computation was also not taken into account for ECDSA-HWMP. IBC-HWMP is the heaviest protocol and the next is ECDSA. SHWMP is less complex than CCMP/IBC because SHWMP encrypts only mutable fields but CCMP encrypts the whole frame body.

To compare the communication overhead of the protocols, the extra amount of data per one frame compared to HWMP was counted for each protocol. To setup the equal security level, 128 bits symmetric encryption, 128 bits hash, and 256 bits elliptic curve were assumed [37]. The communication overhead of CCMP/BIP is 128bit (|CCMP-HDR|+|MIC|=64bit+64bit). SHWMP and IBC-HWMP have 1,152 bits (|MAC|+8|H|=9|H|=128*9) and 512 bits (|G|+|V| = 256*2) overhead, respectively. The overhead of ECDSA is 512 bits (2|Z| = 2*256).

In terms of extra storage requirements compared to HWMP, CCMP and IBC-HWMP require the same amount of storage corresponding to its communication overhead. IBC-HWMP and ECDSA require extra storage to save the pre-computed items. IBC-HWMP and ECDSA require 2048 bits (|G|+|V|+6|Z| ≈8|Z| = 246*8) [38] and 1184bit (4|Z| + |n| = 4*256 +160) [39], respectively. Each node running Watchdog-WHMP has a neighbor monitoring table and keeps track of the packet sent. The monitoring table contains:

- The packet ID (4 bytes)
- The address of the next hop to which the PREQ was forwarded (6 bytes)
- The address of the destination node (6 bytes)
- The expire time (4 bytes)
- TTL (1 byte)
- Hop count (1 byte)
- Metric (4 bytes)

Therefore, Watchdog-WHMP requires (No. of neighbors)*26 bytes of extra storage per node.

### 5.5. One Example of the Security Scheme for IEEE 802.11s

To illustrate how the security requirements (Table 9) and analysis of HWMP Security Protocols (Tables 10 and 12) are utilized in the security protocol design, in this section, we present a simple security scheme.

From Table 9, we can identify that the end-to-end authentication and integrity are common security service requirements for the NMF in the PREQ, PREP, RANN, and PERR frames. A digital signature scheme is a solution satisfying these security requirements. The originator of the messages attaches a digital signature for the NMF of each message. By verifying the signature, not only a destination node but the intermediate nodes on the message transmission path can authenticate the origin of the message and confirm the integrity of NMF. Thus, with these security services for the NMF, we can protect against a path diversion attack and an impersonation attack, as shown in Tables 7 and 8.

From Table 9, we can identify that a forgery detection method is a common security service requirement for MF in the PREQ, PREP and RANN frames. Because the most important information in MF of these messages is the metric, in this example we introduce a new field called the previous node metric (PNM) to detect illegal modification of the metric by an intermediate node. This field carries the metric value of a node one hop ahead to provide to the node one hop behind along the transmission path.

By utilizing the existing IEEE 802.11i key hierarchy protocol with CBC-MAC (cipher block chaining message authentication code) on the MF of the message, the protocol is able to provide point-to-point integrity. For the broadcast messages (PREQ or RANN), GTK will be used to generate the CBC-MAC, while PTK will be used for the unicast message (PREP). The MF of the message will also be encrypted, just as in the current protection mechanism. In short, the current security mechanism can be used to protect the MF alone instead of the entire message.

Equations (17–19) provide the aforementioned security features for PREQ, RANN and PREP, respectively. Enc*_GTK_*(*MF*) and Enc*_PTK_*(*MF*) indicate that the MF will be encrypted using the GTK and PTK, respectively. *CBC*-*MAC_GTK_*(*MF*) and *CBC*-*MAC_PTK_*(*MF*) indicate that the CBC-MAC will be generated by using the GTK and PTK, respectively. *Sign_Pri_* () represents signing with node 's private key. An ID-based signature scheme is useful for the WMN because it does not require public key certificate management and can drive the public key from signer's identity information.

(17) $$\text{PREQ}:{\text{Enc}}_{\text{GTK}}(MF),\text{NMF},\text{CBC}-{\text{MAC}}_{\text{GTK}}(MF),\;{\text{Sign}}_{{\text{Pr}}_{\text{source}}}(\text{NMF}),{\text{Sign}}_{{\text{Pr}}_{i}}(\text{metric}),{\text{Sign}}_{{\mathrm{Pr}}_{i-1}}(\text{PNM})$$

(18) $$\text{RANN}:{\text{Enc}}_{\text{GTK}}(MF),\text{NMF},\text{CBC}-{\text{MAC}}_{\text{GTK}}(MF),\;{\text{Sign}}_{{\text{Pr}}_{\text{source}}}(\text{NMF}),{\text{Sign}}_{{\text{Pr}}_{i}}(\text{metric}),{\text{Sign}}_{{\mathrm{Pr}}_{i-1}}(\text{PNM})$$

(19) $$\text{PREP}:{\text{Enc}}_{\text{PTK}}(MF),\text{NMF},\text{CBC}-{\text{MAC}}_{\text{PTK}}(MF),\;{\text{Sign}}_{{\text{Pr}}_{\text{source}}}(\text{NMF}),{\text{Sign}}_{{\text{Pr}}_{i}}(\text{metric}),{\text{Sign}}_{{\mathrm{Pr}}_{i-1}}(\text{PNM})$$

A source node initiates a PREQ, RANN, or PREP message with a signature for the NMF *Sign*_Pr_*_source_*(NMF) to provide the end-to-end integrity. The source node's public key will then be used to verify the signature at the immediate nodes and the destination node. After receiving *Sign*_Pr_*_i_*(metric) and *Sign*_Pr_*_i_*_-1_(*PNM*), the *_i_*_+_*_1 th_* node then verifies these signatures and checks whether *_metric_*> *PNM* and the difference value is larger than a threshold value. If the verification is successful, it can convince that the value of the node is not forged.

Except for *Enc_GTK_*(*MF*), *Enc_PTK_*(*MF*) and NMF in Equations (17–19), the other message fields can be attached to each message as extended fields, such as SAODV.

## 6. Conclusions

In this paper, the vulnerabilities of the HWMP protocol based on the different types of possible attacks against the protocol were explored. Based on a vulnerability analysis, the security requirements for HWMP were determined. The existing HWMP security protocols, CCMP, BIP, SHWMP, IBC-HWMP, ECDSA-HWMP and Watchdog-HWMP, were analyzed based on these security requirements. We determined that the existing security protocols do not provide sufficient security protection for the HWMP frames.

As shown in our security analysis, CCMP and BIP can protect the HWMP frames from external attacks. In other words, the existing security protocol, as defined in the 802.11w standard, is sufficient to protect the HWMP frames from external attacks. Therefore, the real problem with respect to HWMP security is the possibility of internal attacks. Unfortunately, SHWMP provides insecure end-to-end authentication and integrity for broadcasting and unicasting messages. Additionally, SHWMP does not have any security schemes for PERR HWMP routing protocol frames, and it does not provide the authentication and integrity assurance of NMF from the source node to protect NMF modification attacks. In addition, an anti-replay scheme does not exist in SHWMP.

IBC-HWMP and ECDSA-HWMP attempt to address the problem of internal attacks, but their protection against external attacks is highly flawed. These two schemes have no end-to-end and point-to-point integrity service for NMFs. IBC-HWMP does not explain how to protect RANN and PERR. This protocol does not provide the authentication and integrity assurance of non-mutable field from the source node for the intermediate nodes on the transmission path to protect non-mutable field modification attacks. In addition, an anti-replay scheme does not exist in this protocol. Because the two schemes use a digital signature on the broadcasting PREQ message, a significant number of message drops can occur if the signature verification cannot be processed at line speed. Watchdog-HWMP cannot detect illegal modification or creation in the non-mutable field, which is a major target for internal attacks. As the number of nodes increase, Watchdog-HWMP suffers much more performance degradation in terms of packet loss throughput. It is desirable to include the monitoring of message drop and non-mutable field change into watchdog processing. SHWMP, IBC-HWMP, or ECDSA-HWMP does not provide any permission level for an MP to declare itself as the root mesh STA.

We conclude that CCMP/BIP should always be taken into consideration when developing a new security protocol for HWMP because it is very effective against external attacks. We have the following recommendations for the future development of the HWMP security protocol. First, end-to-end based integrity services for NMF must be provided. These services will prevent intermediate mesh STAs from modifying the sequence number or impersonating originator/target mesh STAs. Second, a permission level should also be implemented so that only authorized mesh STAs can designate themselves as the root mesh STA. Third, an efficient method to detect a fake path metric from any malicious mesh STAs should be implemented. One of the most difficult issues to address is mutable field forgery detection. Because a path metric in MF should be updated by intermediate mesh STAs as it propagates, it cannot be protected with end-to-end authentication and integrity services. Fourth, any security schemes for RANN and PERR must be implemented. We found that the existing security protocols for HWMP do not protect RANN and/or PERR messages. Fifth, the authentication and integrity assurance from the source node for the intermediate nodes on the transmission path should be implemented. The existing security protocols for HWMP do not provide this security service. This service is very important to protect NMF modification or forgery activities related with route disruption, route invasion, resource consumption, and node isolation attacks. Sixth, anti-replay schemes should be implemented. We determined that the use of the sequence number is not sufficient to protect against replay attacks. The sequence number is only effective in avoiding a routing loop.

We have presented a quantitative complexity comparison among the protocols and an example of security scheme for HWMP to demonstrate how the results of our security analysis can be utilized. We hope that our analysis and suggestions will assist future research on HWMP security.

## Figures and Tables

**Table 1.** Routing table for mesh STA C.

**Mesh STA**	**HWMP Sequence Number**	**Next Hop**	**Path Metric**	**Hop Count**	**Lifetime**
MAC A	[MAC A sequence number]	MAC B	[metric AB] + [metric BC]	2	[lifetime]
MAC B	-	MAC B	[metric BC]	1	[lifetime]

**Table 2.** Routing table for mesh STA B.

**Mesh STA**	**HWMP Sequence Number**	**Next Hop**	**Path Metric**	**Hop Count**	**Lifetime**
MAC D	[MAC D sequence number]	MAC C	[metric DC] + [metric CB]	2	[lifetime]
MAC C	-	MAC C	[metric CB]	1	[lifetime]

**Table 3.** Data for creating and updating forwarding information upon receiving PREQ and PREP [3].

**Field of Forwarding Information**	**Received PREQ**		**Received PREP**	
	**Forwarding Information for Transmitter**	**Forwarding Information for Originator Mesh STA**	**Forwarding Information for Transmitter**	**Forwarding Information for Target Mesh STA**
HWMP Sequence Number	Invalid if created, no changes if updated	Originator HWMP Sequence Number	Invalid if created, no changes if updated	Target HWMP Sequence Number
Next Hop	Transmitter mesh STA address	Transmitter mesh STA address	Transmitter mesh STA address	Transmitter mesh STA address
Path Metric	Accumulation of *the initial value of the path metric* with the metric of the link to the transmitter	Accumulation of the value of the PREQ field metric with the metric of the link to the transmitter	Accumulation of the initial value of the path metric with the metric of the link to the transmitter	Accumulation of the value of the PREP field metric with the metric of the link to the transmitter
Number of Hops	1	Hopcount +1	1	Hopcount +1
Lifetime	Stored Lifetime or PREQ Lifetime (whichever is longer)	Stored Lifetime or PREQ Lifetime (whichever is longer)	Stored Lifetime or PREP Lifetime (whichever is longer)	Stored Lifetime or PREP Lifetime (whichever is longer)

Note: the initial value of the path metric is a constant value.

**Table 4.** Misuse of PREQ messages and achievable misuse goals.

**Attacks**	**Route Disruption**	**Route Invasion**	**Node Isolation**	**Resource Consumption**
PREQ drop	Yes (in some case)	No	No	No
PREQ modify and forward	Yes	Yes	Partial	No
PREQ active forge	Yes	Yes	Partial	No

**Table 5.** Misuse of PREP messages and achievable misuse goals.

**Attacks**	**Route Disruption**	**Route Invasion**	**Node Isolation**	**Resource Consumption**
PREP drop	Yes (in some case)	No	No	No
PREP modify and forward	Yes	Yes	No	No
PREP forge reply	Yes	Yes	No	No
PREP active forge	Yes	Yes	No	Yes

**Table 6.** Misuse of PERR messages and achievable misuse goals.

**Attacks**	**Route Disruption**	**Route Invasion**	**Node Isolation**	**Resource Consumption**
PERR drop	Yes(in some case)	No	No	No
PERR modify and forward	Yes	No	No	Yes
PERR active forge	Yes	No	No	Yes

**Table 7.** External attacks, the required security services, and the relevant HWMP frames and fields involved.

**Attacks**	**Descriptions**	**Required Security Services (Point-to-Point)**	**Relevant Frame**	**Involved Field**
Passive Attack	Obtaining important information about the network	Encryption	All	MF NMF
Flooding Attack	Continuously broadcasting frames with false information.	Authentication and integrity	All	All
Replay attack	Replay PREQ messages and spoofing MAC address of victim	Anti-replay scheme	PREO	NMF
Path Diversion Attack	Modifying the path metric.	Authentication and integrity	PREQ PREP RANN	MF
	Modifying the sequence number.	Authentication and integrity	All	NMF
Impersonation Attack	Impersonating a transmitter by changing the transmitter address field.	Authentication and integrity	All	HDR
	Impersonating an originator*/*target by changing the originator/target address field.	Authentication and integrity	All	NMF

**Table 8.** Internal attacks, the required security services, and the relevant HWMP frames and fields involved.

**Attacks**	**Descriptions**	**Required Security Services**	**Relevant Frame**	**Involved Field**
*Path Diversion Attack*	Transmitting frames with fake path metric.	Forgery detection method	PREQ PREP RANN	MF
	Modifying the sequence number.	Authentication and integrity (End-to-end)	All	NMF
*Impersonation Attack*	Pretending to be the root mesh STA.	Root permission level	RANN PREQ	NMF
	Impersonating the originator/target by changing the originator/target address field.	Authentication and integrity (End-to-end)	All	NMF

**Table 9.** Security service requirements according to the relevant frames and fields involved.

**Frame**	**Field**	**Authentication and Integrity (Point-to-point)**	Encryption*(Point-to-Point)	**Authentication and Integrity (End-to-End)**	**Anti-Replay Scheme**	**Root Permission Level**	**Forgery Detection Method**
PREQ	HDR	Need	-	-	-	-	-
	MF	Need	Need	-	-	-	Need
	NMF	Need	Need	Need	Need	Need	-
RANN	HDR	Need	-	-	-	-	-
	MF	Need	Need	-	-	-	Need
	NMF	Need	Need	Need	-	Need	-
PERR	HDR	Need	-	-	-	-	-
	MF	Need	Need	-	-	-	-
	NMF	Need	Need	Need	-	-	-
PREP	HDR	Need	-	-	-	-	-
	MF	Need	Need	-	-	-	Need
	NMF	Need	Need	Need	-	-	-

* indicates optional requirements.

**Table 10.** Security services provided by CCMP and BIP.

**Frame**	**Field**	**Authentication and integrity (Point-to-point)**	Encryption* (Point-to-point)	**Authentication and integrity (End-to-end)**	**Anti-replay scheme**	**Root Permission Level**	**Forgery Detection Method**	
PREQ	HDR	Yes	-	-		-	-	
	MF	Yes	Yes	-		-	No
	NMF	Yes	Yes	No	No	No	-
RANN	HDR	Yes	-	-		-	-	
	MF	Yes	Yes	-		-	No
	NMF	Yes	Yes	No		No	-
PERR	HDR	Yes	-	-		-	-	
	MF	Yes	Yes	-		-	-
	NMF	Yes	Yes	No		-	-
PREP	HDR	Yes	-	-		-	-	
	MF	Yes	Yes	-		-	No
	NMF	Yes	Yes	No		-	-

* indicates optional requirements.

**Table 11.** Analysis of the security services provided by SHWMP based on the security service requirements.

**Frame**	**Field**	**Authentication and integrity (Point-to-point)**	Encryption* (Point-to-point)	**Integrity (End-to-end)**	**Anti-replay scheme**	**Root Permission Level**	**Forgery Detection Method**
PREQ	HDR	No	-	-	-	-	-
	MF	Yes	No	-	-	-	No
	NMF	No	Yes	No	No	No	-
RANN	HDR	No	-	-	-	-	-
	MF	Yes	No	-	-	-	No
	NMF	No	Yes	No	-	No	-
PERR	HDR	No	-	-	-	-	-
	MF	No	No	-	-	-	-
	NMF	No	No	No	-	-	-
PREP	HDR	No	-	-	-	-	-
	MF	Yes	No	-	-	-	No
	NMF	No	Yes	No	-	-	-

* indicates optional requirements.

**Table 12.** Security services provided by IBC-HWMP and ECDSA-HWMP.

**Frame**	**Field**	**Authentication And Integrity (Point-To-Point)**	Encryption* (Point-To-Point)	**Authentication And Integrity (End-To-End)**	**Anti-Replay Scheme**	**Root Permission Level**	**Forgery Detection Method**
PREQ	HDR	No	-	-	-	-	-
	MF	Yes	No	-	-	-	Yes
	NMF	No	No	No	No	No	-
RANN	HDR	No	-	-	-	-	-
	MF	No	No	-	-	-	No
	NMF	No	No	No	-	No	-
PERR	HDR	No	-	-	-	-	-
	MF	No	No	-	-	-	-
	NMF	No	No	No	-	-	-
PREP	HDR	No	-	-	-	-	-
	MF	YES	No	-	-	-	Yes
	NMF	No	No	No	-	-	-

* indicates optional requirements.

**Table 13.** The Comparison of computational complexity for Secure HWMP protocols. The numbers of computation are only counted for one hop transmission.

**Protocol**	**Frame**	**Computational Cost**	**Communication Overhead**	**Additional Storage Requirement**
CCMP/BIP	PREQ	4Ec/2Ec	|CCMP-HDR|+|MIC|/|MMIC|+|MIC|	|CCMP-HDR|+|MIC|/|MMIC|+|MIC|
	PREP	4Ec/2Ec	|CCMP-HDR|+|MIC|/|MMIC|+|MIC|	|CCMP-HDR|+|MIC|/|MMIC|+|MIC|
	RANN	4Ec/2Ec	|CCMP-HDR|+|MIC|/|MMIC|+|MIC|	|CCMP-HDR|+|MIC|/|MMIC|+|MIC|
	PERR	4Ec/2Ec	|CCMP-HDR|+|MIC|/|MMIC|+|MIC|	|CCMP-HDR|+|MIC|/|MMIC|+|MIC|
SHWMP	PREQ	2Ec+46H	|MAC| + 8|H|	|MAC| + 8|H|
	PREP	2Ec+26H	|MAC|+5H	|MAC|+5H
	RANN	2Ec+26H	|MAC|+5H	|MAC|+5H
IBC-HWMP	PREQ	2E+1M+2P	|G|+|V|	|G|+|V|+6|Z|
	PREP	2E+1M+2P	|G|+|V|	|G|+|V|+6|Z|
ECDSA-HWMP	PREQ	1MI+2H+2M+1A	2|Z|	4|Z|+|n|
	PREP	1MI+2H+2M+1A	2|Z|	4|Z|+|n|
Watchdog- HWMP	PREQ			(No. of neighbors)*26 bytes

‖ : size of an item, G : additive field for pairing input, V : multiplicative field for paring output, Z : base field for elliptic curve, n : order n of a base elliptic point.
